# Specifications of the ACMG/AMP variant curation guidelines for the analysis of germline *PALB2* sequence variants

**DOI:** 10.1016/j.ajhg.2025.08.020

**Published:** 2025-09-17

**Authors:** Marcy E. Richardson, Megan F.H. Bishop, Megan A. Holdren, Miguel de la Hoya, Amanda B. Spurdle, Sean V. Tavtigian, Terra Brannan, Colin C. Young, Lauren Zec, Susan Hiraki, Clare Turnbull, Marc Tischkowitz, Kara A. Bernstein, Jean-Yves Masson, Shannon M. McNulty, Tina Pesaran, Alvaro N. Monteiro, Logan C. Walker, William D. Foulkes, Fergus J. Couch

**Affiliations:** 1 Ambry Genetics, Aliso Viejo, CA, USA; 2 Division of Laboratory Genetics and Genomics, Department of Laboratory Medicine and Pathology, Mayo Clinic, Rochester, MN, USA; 3 Molecular Oncology Laboratory, Hospital Clínico San Carlos, IdISSC (Instituto de Investigación Sanitaria del Hospital Clínico San Carlos), 28040 Madrid, Spain; 4 Population Health, QIMR Berghofer Medical Research Institute, Brisbane, QLD 4006, Australia; 5 University of Utah School of Medicine, Salt Lake City, UT, USA; 6 Natera, Inc. 201 Industrial Boulevard, San Carlos, CA, USA; 7 GeneDx, Gaithersburg, MD, USA; 8 Division of Genetics and Epidemiology, Institute of Cancer Research, London, UK; 9 Department of Genomic Medicine, National Institute for Health Research Cambridge Biomedical Research Centre, University of Cambridge, Cambridge, UK; 10 Department of Biochemistry and Biophysics, University of Pennsylvania School of Medicine, Philadelphia, PA 19104, USA; 11 CHU de Québec-Université Laval, Québec, QC, Canada; 12 Department of Pathology and Laboratory Medicine, The University of North Carolina at Chapel Hill, Chapel Hill, NC, USA; 13 Moffitt Cancer Center, Tampa, FL 33612, USA; 14 Department of Pathology and Biomedical Science, University of Otago, Christchurch, New Zealand; 15 Departments of Human Genetics, McGill University, Montreal, QC, Canada; 16 Department of Laboratory Medicine and Pathology, Mayo Clinic, Rochester, MN, USA

**Keywords:** ACMG/AMP guidelines, variant interpretation, classification, PALB2, hereditary breast ovarian and pancreatic cancer, Variant Curation Expert Panel, VCEP

## Abstract

Interpretation of genetic variants is most accurate when gene- and disease-specific considerations are considered. The 2015 ACMG/AMP guidelines form the basis for the application of variant interpretation criteria for Mendelian disorders. The Hereditary Breast, Ovarian, and Pancreatic Cancer Variant Curation Expert Panel (HBOP VCEP) has undertaken the process for creating gene- and disease-specific specifications for the interpretation of *PALB2* germline sequence variants. The HBOP VCEP is comprised of experts in the fields of clinical and molecular genetics, epidemiology, functional assays, and variant interpretation. The group met regularly to consider each of the codes from the 2015 ACMG/AMP guidelines to determine their relevance for *PALB2*. After criteria were created using database analysis, literature review, and expert opinion, they were vetted against a diverse set of pilot variants and ultimately finalized. The HBOP VCEP advised against using 13 codes, limited the use of six codes, and tailored nine codes to create the final *PALB2* variant interpretation guidelines. Among the 39 pilot variants, 37 were in ClinVar, and using the new specifications concordant classifications resulted for 31 of the variants (84%). Of the 14 variants of uncertain significance/conflicting variants in ClinVar, four were classified by the VCEP, likely due to code combination modifications and refined population frequency cutoffs. The *PALB2*-specific guidelines put forward by the HBOP VCEP represent a conservative approach to classifying variants in *PALB2* and lead to improved classifications relative to current ClinVar entries. Adoption of these specifications will help to harmonize classifications deposited in the public domain.

## Graphical abstract

**Figure:**
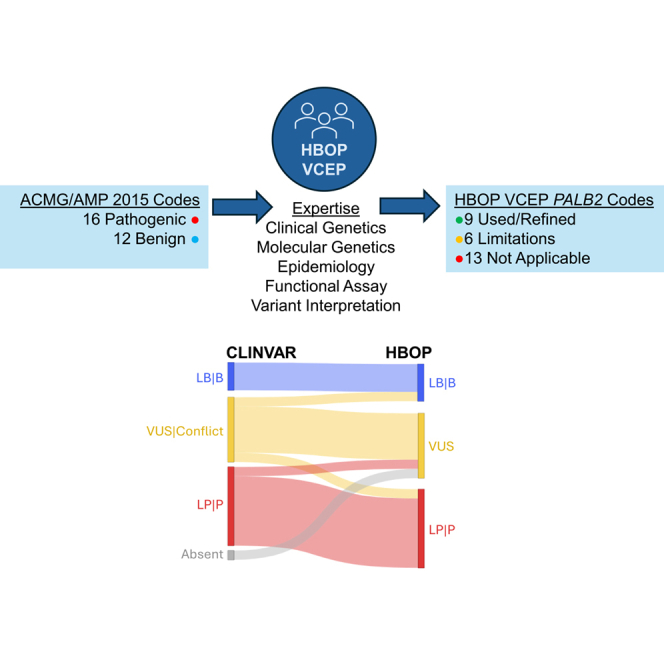

## Teaser

The HBOP VCEP developed *PALB2*-specific ACMG/AMP variant interpretation guidelines by tailoring, limiting, or removing existing codes. Testing on 39 pilot variants improved concordance with ClinVar and reclassified several variants of uncertain significance. These conservative, evidence-based specifications will support harmonized, accurate *PALB2* variant classification in the public domain.

## Introduction

The American College of Medical Genetics and Genomics and the Association for Molecular Pathology (ACMG/AMP) published a joint paper establishing a set of guidelines for the interpretation of germline variants with Mendelian inheritance patterns.^1^ These guidelines serve as the foundation for general variant interpretation. However, as each gene and disease has nuances, the guidelines need to be tailored to accurately interpret variants in a gene-specific manner. It is the purview of Variant Curation Expert Panels (VCEPs), under the guidance of the Sequence Variant Interpretation group (SVI), to assemble a group of experts to refine and vet gene-specific guidelines for the purpose of ongoing variant curation.^2^ Due to its rigor and comprehensiveness, the assembly and training of a VCEP and the undertaking and documenting of its activities is a Food and Drug Administration (FDA)-approved process that is respected within the genetics community.

The 2015 iteration of the ACMG/AMP variant interpretation guidelines makes use of multiple evidence types including clinical, computational/predictive, population-based, and functional data. These data are parsed into 28 codes, which can each be variably weighted as supporting, moderate, strong, or very strong based on the robustness of the data. The weighted codes are compiled to form an overall five-tiered interpretation as pathogenic (P), likely pathogenic (LP), variant of uncertain significance (VUS), likely benign (LB), or benign (B).

The Hereditary Breast, Ovarian, and Pancreatic Cancer (HBOP) VCEP was formed in 2018 with a goal of defining gene-specific variant interpretation guidelines for *ATM* (Hugo Gene Nomenclature Committee [HGNC]:795, MIM: 607585), *BARD1* (HGNC:952, MIM: 601593), *BRIP1* (HGNC:204763, MIM: 605882), *CHEK2* (HGNC:16627, MIM: 604373), *PALB2* (HGNC:26144, MIM: 610355), *RAD51C* (HGNC:9820, MIM: 602774), and *RAD51D* (HGNC:9823, MIM: 602954) (https://clinicalgenome.org/affiliation/50039/). The HBOP VCEP comprises an international group of experts in the fields of clinical genetics, variant interpretation, and medical and molecular research. The HBOP VCEP develops specifications in a sequential manner and has finalized specifications for Ataxia Telangiectasia Mutated (*ATM*),^3^ and it now puts forward specifications for “Partner And Localizer of BRCA2,” *PALB2*.

*PALB2* (MANE Select GenBank: NM_024675.3) is located on chromosome 16p12.2 and contains 13 exons encoding 1,186 amino acids.^4^ It is a tumor-suppressor gene acting in the homologous recombination repair pathway, which is responsible for repairing DNA double-strand breaks and maintaining genome integrity.^5^ In the heterozygous state, germline pathogenic variants (PVs) cause *PALB2*-related cancer predisposition (MONDO:0016419; MIM: 613348 Pancreatic Cancer, Susceptibility To, 3; and MIM: 620442 Breast-Ovarian Cancer, Familial, Susceptibility To, 5; Brovca5) leading to increased lifetime risk of developing breast, ovarian, and pancreatic cancer but with incomplete penetrance for these cancers.^6^ In the compound heterozygous or homozygous state, PVs in *PALB2* cause Fanconi anemia (FA). Bi-allelic pathogenic variants in *PALB2* specifically cause FA subtype N (FA-N, MONDO:0012565, MIM: 610832 Fanconi Anemia, Complementation Group N; FANCN), which is similar to other subtypes of FA caused by bi-allelic PVs in other genes. FA is a severe condition characterized by growth retardation, congenital malformations, and pediatric malignancies.^7^^,^^8^

The identification of LP/P variants in *PALB2* affords carriers access to increased surveillance and risk reducing measures according to clinical guidelines. In rare situations where both partners are PV carriers, they may consider additional family planning measures given the association of bi-allelic *PALB2* PVs with a severe disease. Therefore, the accurate interpretation of variants is crucial for carriers of *PALB2* germline pathogenic variants. Here, we describe the process of forming the HBOP VCEP, the evidence code modifications for *PALB2*, and the outcomes of the pilot curation effort.

## Methods

### ClinGen HBOP VCEP

The HBOP VCEP is comprised of 23 members from seven countries with a wide range of expertise, including basic science research, variant interpretation, tumor pathology, clinical genetics, molecular genetics, population science, and genetic counseling. The membership convenes virtually on a monthly basis. The HBOP VCEP has been addressing genes sequentially based on perceived priority need by the variant interpretation community. Initial specifications were drafted based on consideration of literature, internal data, and expert opinion. In some instances, where gene and disease states overlap with *BRCA1* (HGNC:1100; MIM: 113705) and *BRCA2* (HGNC:1101; MIM: 600185), codes were jointly considered with the ENIGMA BRCA1 and BRCA2 VCEP (https://clinicalgenome.org/affiliation/50087/).

### Pilot phase

These initial specifications were tested against a list of 39 *PALB2* variants with consideration of a variety of variant types, evidence codes applied, and ClinVar interpretations. Relevant clinical and allelic data from unpublished sources were solicited from the membership ahead of curation. Variants were curated by two trained biocurators, and the codes applied were compared between the two curations. Consensus curations were harmonized and deposited into the Variant Curation Interface (VCI).^9^ Classifications followed the original five-tier model (B, LB, VUS, LP, and P) and evidence code combinations, with a few modifications that are supported by a Bayesian framework.^10^

### Final *PALB2* specifications

Refinements made in response to the pilot round of interpretations were submitted to the SVI for review. The final round of refinements, as recommended by the SVI, was implemented and resubmitted for approval, which was granted on March 17, 2023. Pilot variants were deposited to ClinVar with 3-star status. The most recent *PALB2* guidelines will be found on the Criteria Specification Registry, as specifications will be updated as this VCEP makes future modifications (https://cspec.genome.network/cspec/ui/svi/doc/GN077).

## Results

### Codes limited or not applicable for *PALB2*: PS1 missense; PS2, PS3, and PS4 proband counting; PM1, PM4, and PM5 missense; PM6, PP2, and PP3 missense; PP4, PP5, and BS2 autosomal dominant disease; BS3, BP2, BP3, and BP4 missense; BP5; and BP6

There are 28 codes in the 2015 ACMG/AMP guidelines for interpretation of sequence variants: 12 toward benign and 16 toward pathogenic. The HBOP VCEP does not recommend the use of 13 of these codes under any circumstance and recommends limiting the application of a further six codes to specific situations, as detailed below and in Table 1.

**Table 1:** Summary of PALB2-specific rule specifications

**Code**^a^	**Original application**	***PALB2*-modified application**
PVS1	null variant in a gene where loss of function is a known mechanism of disease	per *PALB2* exon map (Figure 1) and *PALB2* PVS1 decision tree (Figure 2) • PVS1_Variable: predicted splice defect • PVS1_Variable(RNA): observed splice defect • note: PVS1 and PVS1(RNA) have code combination restrictions: see Table 3
USED		
PS1	same amino acid change as a previously established pathogenic variant regardless of nucleotide change	• missense: do not use. Missense changes are not yet confirmed as a mechanism of disease for *PALB2* • splicing: (use as PS1_ Variable) per SVI guidelines: see PS1 table (Table 2)
LIMITED		
PS2	*de novo* (paternity confirmed) in an individual with the disease and no family history	do not use for autosomal dominant or autosomal recessive disease
NOT USED		
PS3	Well-established *in vitro* or *in vivo* functional studies supportive of a damaging effect	do not use: lack of known pathogenic missense variants for validation
NOT USED		
PS4	the prevalence of the variant in affected individuals is significantly increased compared with the prevalence in controls	• do not use for proband-counting studies • case-control studies; *p* value ≤0.05 AND (odds ratio, hazard ratio, or relative risk ≥3 OR lower 95% CI ≥1.5)
LIMITED		
PM1	located in a mutational hotspot and/or critical and well-established functional domain	do not use: missense pathogenic variation in *PALB2* is not yet confirmed as a mechanism of disease
NOT USED		
PM2	absent/rare from controls in an ethnically matched cohort population sample	• variant absent in gnomAD v.2.1.1 or present in ≤0.0003% in all sub-populations • exception: under-represented subpopulations with *n* = 1 but frequency >0.0003% • not considered a conflicting piece of evidence for variants that otherwise are likely benign/benign • use as PM2_Supporting (not moderate)
USED		
PM3	for recessive disorders, detected *in trans* with a pathogenic variant	per Fanconi anemia PM3/BP2 tables (Table 4 and Box 1)
USED		
PM4	protein length changes due to in-frame deletions/insertions in a non-repeat region or stop-loss variants	• do not use for in-frame deletions/insertions that are not already PVS1 eligible: no information is available to justify the application of this rule. In addition, missense/small in-frame indels are not yet confirmed as a mechanism of disease for *PALB2* • do not use for stop loss due to lack of data on stop-loss variants
NOT USED		
PM5	missense change at an amino acid residue where a different missense change determined to be pathogenic has been seen before	• missense: do not use. Missense changes are not yet confirmed as a mechanism of disease for *PALB2* • apply as PM5_Supporting to frameshifting or truncating variants with premature termination codons upstream of His1184, based on location of the most C-terminal known pathogenic variant, p.Tyr1183Ter • apply to splice variants as PM5_supporting for splice variants can only be applied for variants premature termination codons upstream of His1184 where PVS1_VS(RNA) is applied based on high-quality observed splicing impact and must be prone to nonsense-mediated decay
LIMITED		
PM6	confirmed *de novo* without confirmation of paternity and maternity	do not use for autosomal dominant or autosomal recessive disease
NOT USED		
PP1	co-segregation with disease in multiple affected family members	• autosomal dominant condition • PP1_Strong: LOD ≥ 1.26 or Bayes factor (LR) ≥18:1 • PP1_Moderate: LOD ≥ 0.60 or Bayes factor (LR) ≥4:1 • PP1: LOD ≥ 0.3 or Bayes factor (LR) ≥2:18 • autosomal recessive condition: informative instances of co-segregation in Fanconi anemia families are too rare to be formally analyzed at this time; however, this VCEP supports approaching this similarly to the ITGA2B/ITGB3 and hearing loss VCEPs who have outlined PP1 criteria for these autosomal recessive disorders
USED		
PP2	missense variant in a gene that has a low rate of benign missense variation and where missense variants are a common mechanism of disease	do not use. Missense is not yet confirmed or refuted as a mechanism of disease for *PALB2*
NOT USED		
PP3	multiple lines of computational evidence support a deleterious effect on the gene or gene product	• missense: do not use. Published predictors have yet to predict functional outcome for *PALB2* missense variants • splicing: SpliceAI score ≥0.2 • do not use in conjunction with PVS1(RNA) • use caution in applying the wrong type of computational evidence (missense vs. splicing) toward the cumulative body of evidence for the opposite mechanism
LIMITED		
PP4	phenotype specific for disease with single genetic etiology	• do not use for autosomal dominant disorder • for autosomal recessive disorder, see PM3 for specific phenotype considerations (Table 4)
LIMITED		
PP5	reputable source recently reports variant as pathogenic, but the evidence is not available to the laboratory to perform an independent evaluation	do not use
NOT USED		
BA1	allele frequency is above 5% in Exome Sequencing Project, 1000 Genomes, or ExAC	>0.1% (0.001)
USED		
BS1	allele frequency is greater than expected for disorder	>0.01% (0.0001)
USED		
BS2	observed in a healthy adult individual for a dominant (heterozygous) disorder with full penetrance expected at an early age	• autosomal dominant condition: do not use—*PALB2* has incomplete penetrance and causes common cancer phenotypes frequently subject to phenocopy • autosomal recessive condition: per Fanconi anemia PM3/BS2 tables (Table 4)
LIMITED		
BS3	well-established *in vitro* or *in vivo* functional studies show no damaging effect on protein function	do not use: lack of known pathogenic missense variants for validation
NOT USED		
BS4	lack of segregation in affected members of a family	• autosomal dominant condition • BS4_Supporting: LOD ≤ −0.32 or Bayes factor (LR) ≤0.48 • BS4_Moderate: LOD ≤ −0.64 or Bayes factor (LR) ≤0.23 • BS4: LOD ≤ −1.28 or Bayes factor (LR) ≤0.053:1 • autosomal recessive condition: informative instances of non-segregation in Fanconi anemia families are too rare to be formally analyzed at this time; however, this VCEP supports approaching this similarly to the ITGA2B/ITGB3 and hearing loss VCEPs who have outlined PP1 criteria for these autosomal recessive disorders
USED		
BP1	missense variant in gene where only loss of function causes disease	apply to all missense variants
USED		
BP2	observed in *trans* with a pathogenic variant for a fully penetrant dominant gene/disorder	do not use: see BS2 related bi-allelic individuals not affected by Fanconi anemia
NOT USED		
BP3	in-frame deletions/insertions in a repetitive region without a known function	do not use
NOT USED		
BP4	multiple lines of computational evidence suggest no impact on gene or gene product	• missense/in-frame: do not use. Published predictors have yet to predict functional outcome for *PALB2* missense variants • splicing: SpliceAI score ≤0.1 • do not use in conjunction with BP7_Variable(RNA) • use caution in applying the wrong type of computational evidence (missense vs. splicing) toward the cumulative body of evidence for the opposite mechanism
LIMITED		
BP5	variant found in an individual with an alternate molecular basis for disease	do not use
NOT USED		
BP6	reputable source recently reports variant as benign, but the evidence is not available to the laboratory to perform an independent evaluation	do not use
USED		
BP7	a synonymous (silent) variant for which splicing prediction algorithms predict no impact to the splice consensus sequence nor the creation of a new splice site AND the nucleotide is not highly conserved	BP7: synonymous and deep intronic • can be used for deep intronic variants beyond (but not including) +7 (donor) and −21 (acceptor) • may also apply BP4 to achieve likely benign • is not considered a conflicting piece of evidence against a body of evidence supporting a pathogenic splice defect BP7_Variable(RNA): RNA functional studies
USED		

VCEP, Variant Curation Expert Panel.

a Table is based on ACMG/AMP rules from Richards et al.^1^

#### Common phenotype, do not apply: PS4 proband counting, PP4, BS2 autosomal dominant, and BP2

In contrast to severe childhood diseases, breast cancer and, to a lesser degree, ovarian and pancreatic cancer are relatively common with later ages at onset. Although these cancers have multiple genetic etiologies, the vast majority are sporadic in nature.^11^ There is currently no pathology, histology, or other biomarker that can confidently distinguish hereditary from sporadic breast and pancreatic cancers. As such, phenotype data have limited use in variant interpretation for *PALB2-*related cancer predisposition in the absence of models requiring large datasets for statistical power.^12^ This prompted the HBOP VCEP to recommend against using codes that heavily rely on phenotype: PS4 (autosomal dominant proband counting), PP4 (phenotype related to a single genetic etiology), BP2 (in *trans* observations in a healthy individual), and BS2 (variant identified in a healthy adult). Of note, BP2 and BS2 have similar language in the context of a person carrying bi-allelic *PALB2* PVs who is *not* affected by FA. To harmonize with the BRCA1/2 VCEP, we have adopted the code BS2 for this observation and, therefore, nullify the use of BP2 at all for *PALB2*.^13^

#### Missense pathogenicity is not established, do not apply: PS1 missense; PS3/BS3, PM1, and PM4 in frame; PM5 missense; PP3/BP4 missense; and BP3

An additional feature of *PALB2* that prevents the application of some codes is that, to date, missense variation and one-amino-acid, in-frame insertion or deletion events are not yet established as a mechanism of pathogenicity. Although there are some indications that missense PVs exist, they are believed to be rare. There are currently four functional studies that have evaluated multiple missense variants using impact on homology-directed DNA repair.^14^^,^^15^^,^^16^^,^^17^ However, the validation of such studies using the SVI-sanctioned methodology becomes challenging given the circular problem of not having missense PVs with which to benchmark the assays.^18^ Although the SVI-sanctioned functional assay validation protocol permits the use of a supporting line of evidence for established and well-accepted functional studies, this VCEP has elected to withhold the application of functional weight until missense variants can be clinically tied to pathogenicity. The empirical link between missense dysfunction and clinical relevance will require high-throughput variant functional results as, given the expectation of a low number of non-functional missense variants and a paucity of carriers for these variants, it is expected that a pooled case-control analysis will be required to make this link. Given this missing link, the HBOP VCEP has limited the use of the following missense-driven codes: PS1 (protein: same amino acid substitution as previously established LP/P variant); PM1 (regional mutational hotspot); PM4/BP3 (one-amino-acid, in-frame insertion or deletion events); PM5 (missense change at an amino acid hotspot); and PS3/BS3 (protein function). Similarly, the HBOP VCEP has also limited the use of PP3 and BP4, the codes ascribed to *in silico* predictions of missense variants. Of note, PM5 for protein observations is limited; however, the VCEP is using this code at a supporting line of evidence to bolster PVS1 and PVS1(RNA) evidence (see PM5 below). Also of note, the PS1 code is not applied for protein observations but is still used for RNA observations (see PS1 below).

#### Lack of situational observation, do not apply: PS2, PM6, and PM4 stop loss

The HBOP VCEP has limited the use of several codes due to the dearth of evidence to support weighting them. A variant that is acquired in the germline (rather than inherited) in an individual with pathognomonic condition is strong evidence of pathogenicity. The use of *de novo* codes PS2 and PM6 is not recommended for *PALB2*, however, as to our knowledge there have been no observances of *de novo PALB2* variants in a bi-allelic state. In addition, a *de novo* occurrence in an individual with cancer who has a heterozygous variant would not be informative, given that *PALB2*-related cancer types are common rather than pathognomonic. Therefore, such observations are not informative for variant classification.

Similarly, there are not enough observances to confidently apply PM4 for stop-loss variants. Interestingly, all three reading frame registers for stop loss or extensions would result in the inclusion of five or fewer amino acids to PALB2. The predicted impact of these additional amino acids is elusive given the current methods of structural and functional modeling.

#### General non-applicability, do not apply: PP2, BP3, BP5, and PP5/BP6

Lastly, there is evidence that missense variation is largely tolerated in PALB2 (gnomAD v.4.1.0 missense *Z* score 0.82: https://gnomad.broadinstitute.org/gene/ENSG00000083093?dataset=gnomad_r4, accessed June 12, 2025). Therefore, the PP2 code (missense constraint) should not be applied to this gene. Additionally, *PALB2* does not have any repeat regions where in-frame insertions and deletions may be considered tolerated, making the BP3 code obsolete. BP5 ascribes benign evidence when a proband is found to have a different molecular basis for disease. However, since many individuals are double heterozygous for PVs in other HBOP/HBOC genes without notably different phenotypes, BP5 is not applied. There is evidence that BP5 could be applied if datasets are carefully calibrated; however, we cannot recommend this as a generalizable code.^19^ Finally, as they are already limited by the SVI, the use of classifications from reputable sources without additional detailed information is not used in the PALB2-specific guidelines: PP5 and BP6 are not used.^20^

### Codes applied or with limited application

#### Population-based specifications: BA1, BS1, and PM2_Supporting

##### BA1 and BS1

BA1 and BS1 indicate that a variant is too frequent in the general population to be disease causing. To calculate thresholds for BA1 (standalone benign) and BS1 (strong benign), the HBOP VCEP used the Whiffin-Ware equation, which incorporates the mode of inheritance, the allelic heterogeneity, the genetic heterogeneity, and the penetrance of a given associated disorder.^21^ For *PALB2*, to be conservative, the prevalence of breast cancer (one in eight women) was used in this equation. Genetic heterogeneity, which is the proportion of breast cancer attributable to *PALB2*, was conservatively set to 1% (0.01). Allelic heterogeneity, which is the proportion of disease that is attributable to a single variant, was conservatively set to 100% (1.0) for BA1 and 10% (0.1) for BS1. Using a penetrance of 53% (0.53),^6^ the maximum credible allele frequency was 0.118% for BA1 and 0.0118% for BS1. For simplicity, these values were rounded to 0.1% for BA1 and 0.01% for BS1.

##### PM2

PM2 is a code indicating that a variant is considered very rare in the population, which is usually a prerequisite for a PV. As per the SVI’s later guidance put forth as a white paper (https://clinicalgenome.org/site/assets/files/5182/pm2_-_svi_recommendation_-_approved_sept2020.pdf) the PM2 rule is only used as a supporting line of pathogenic evidence (PM2_Supporting). Given the rarity of genetic variation in *PALB2* in general,^22^ PM2_Supporting may be applied to variants with a frequency of 0.0000033 (≤1/300,000 alleles) with at least 30× coverage at the locus.^23^ Of note, since most benign variation is also rare, PM2_Supporting is not considered a conflicting piece of evidence when all other evidence points toward a variant being benign. Most PVS1-eligible variants will be classified as at least LP based on additional evidence from either PM5_Supporting and/or PM2_Supporting (see Box 2).

#### Loss-of-function codes: PVS1 and PVS1(RNA)

**Figure 1:**
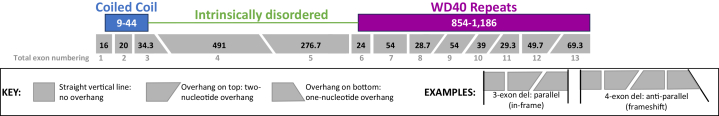
*PALB2* exon numbering and reading frame *PALB2* is depicted exon by exon. The number of amino acids encoded by each exon is depicted within the boxes in black text. The two major functional domains are labeled above the exons in blue (coiled-coil domain) and purple (WD40 repeat domain) boxes. Each exon is shaped to indicate the number of overhanging nucleotides at either end, which will assist in determining any reading-frame changes from gross deletions or duplications of whole single or multiple exons. A vertical line indicates a blunt start or end with no overhanging nucleotides. An upper overhang on either side represents a two-nucleotide overhang; a lower overhang represents a single-nucleotide overhang on that side. To use this diagram, a line drawn at the start and end of a deletion or duplication will be either parallel (in-frame event) or non-parallel (frameshift) as in the examples.

Loss of function (LoF) is the mechanism of disease for *PALB2*, and PVS1 is a very strong line of evidence toward pathogenicity based on the expectation that a LoF variant will cause nonsense-mediated decay (NMD) and/or adversely affect critical functional domains. Such variants include start-loss, nonsense, frameshifting indels, gross deletions/duplications (also called structural variation), and canonical splice site variants at the first and last two nucleotides of an intron (±1,2). The VCEP defined *PALB2-*specific guidelines to inform the application of PVS1 as it pertains to the updated guidance published by the SVI in 2018.^24^ This guidance considers the following features and is based on transcript GenBank: NM_024675.3, which is depicted in Figure 1.

##### PVS1

(1) Start codon: the annotated start codon is considered PVS1 eligible because the closest alternative in-frame methionine is at Met296 in exon 4, the use of which would result in loss of the coiled-coil domain, which is considered critical to protein function.^25^ In addition, c.395delT (p.Val132AlafsTer43) and c.757_758delCT (p.Leu253IlefsTer3), both of which originate upstream of nucleotide 886 (Met296) are identified in individuals with FA-N,^7^ supporting the use of the annotated Met1 as the start codon.
(2) Naturally occurring alternative splicing: several naturally occurring alternative splicing isoforms have been described. Yet, after careful examination, none are considered a candidate rescue transcript, as they demonstrate very low levels of expression and/or are missing sequences considered to be critical to protein function (see functional domains below). Therefore, all presumed LoF events are considered to occur in biologically relevant transcripts.^26^ This feature also influences the application of PM5_Supporting.
(3) Functional domains: PALB2 is comprised of a WD40 beta propeller (WD40) and coiled-coil domain (CC) separated by an intrinsically disordered region (UniProt: Q86YC2 and Figure 1). The WD40 domain is considered indispensable for clinically relevant PALB2 function based on the following evidence. (1) The WD40 domain interacts with many different protein partners that are involved in the double-strand break repair pathway.^27^ (2) Splice variants that lead to in-frame (NMD-escaping) losses impacting the WD40 domain are found in *trans* with LoF *PALB2* variants in individuals with FA: *PALB2* c.3113+5G>C with *PALB2* c.395delT (p.Val132AlafsTer45) and *PALB2* c.3350+4A>G with *PALB2* c.2393_2394insCT (p.Thr799LeufsTer53).^7^ Therefore, LoF variants that are predicted to escape NMD but that adversely affect the WD40 domain are assigned PVS1 at the very strong weight. Of note, because of the strong evidence described herein, this is upweighted from the original guidance of PVS1_Strong for in-frame variants impacting an important domain.^24^ For the region between amino acids 45 and 853, splicing and structural variants with in-frame effects receive default strengths of strong for impacts >10% and moderate for impacts <10%. However, it is possible that, due to the intrinsically disordered nature of this region, in-frame effects may in fact be tolerated.
(4) C-terminal boundary: two different C-terminal truncating variants (*PALB2* c.3549C>A [p.Tyr1183Ter] and *PALB2* c.3549C>G [p.Tyr1183Ter]) were identified in *trans* with *PALB2* LoF variants in three unrelated individuals with FA (FA-N)^7^ (see section PM3). Both variants cause an NMD-escaping transcript that is predicted to cause the same protein change, p.Tyr1183Ter, which preserves all but the last three amino acids of the PALB2 protein, supporting a critical role for Tyr1183. The PALB2 WD40 toroidal structure is “sealed” in the seventh blade by interaction of the C-terminal strand with the incomplete N-terminal blade. The last four residues of PALB2 (Tyr1183, His1184, Tyr1185, and Ser1186) are directly involved in this interaction (molecular Velcro hydrogen bonding).^28^ However, excepting Tyr1183, there is currently no evidence that any of these residues are clinically relevant, setting this residue as the C-terminal boundary such that any variant truncating the PALB2 protein upstream of His1184 is ascribed PVS1 (Table S1).
(5) Splicing predictions/observations: canonical splice sites, at the first and last two nucleotides of an intron (±1,2) and, to a lesser degree, last-nucleotide-of-exon variants, have a high probability of having a substantial splice defect. However, many factors will inform the weight afforded to such any predicted or observed splice effect, including (1) whether the event is complete or incomplete, (2) whether the event is frameshifting or in-frame, (3) whether a frameshifting event is NMD prone or escaping, and (4) whether an in-frame or NMD-escaping event impacts a critical or inconsequential part of the protein. This VCEP systematically collected and reviewed predictions and observed splice effects for each canonical ±1,2 and last-nucleotide position and ascribed a PVS1 weight based on those results, which are displayed in Table S1. Of note, the HBOP VCEP recommends applying the PVS1 code to the last-nucleotide variants based on the high degree of conservation at the last-nucleotide position of U2 donor sites. This should be applied at one level lower than the baseline PVS1 weight afforded to the neighboring +1/2 positions and only in a scenario where the intronic portion of the donor site does *not* conform to the consensus sequence of gtrrgt (where r is either adenine or guanine). In a situation where the intronic portion *does* conform to the consensus sequence, a last nucleotide substitution may be tolerated, and PP3 or BP4 weight should be applied as appropriate.

##### PVS1_Variable(RNA)

The HBOP VCEP distinguishes a predicted from an observed splice defect. As such, it has co-opted the use of PVS1 by applying the extension (RNA) to describe an observed splice defect. This code shall be applied to all observed splice defects regardless of whether it occurs at a canonical position (±1,2) or other position including deeper intronic and exonic variants as affected by any variant type, including synonymous, nonsense, missense, or frameshift. The weight of the observed splice defect can be modified based on the quality of the assay, including such features as (1) whether human-derived material (preferred) or *in vitro* minigene is used, (2) the use of NMD inhibitors in a cell line where NMD would be expected to occur, (3) primer design (e.g., to capture major alternative isoforms or multi-cassette events), (4) quantification methodology (e.g., SNP analysis [preferred], capillary electrophoresis, and RT-qPCR), and (5) the degree of splice defect (e.g., complete or incomplete). Additional guidance from the SVI is available.^29^ It is worth including here that the application of evidence for an observed lack of splice effect is coded as BP7(RNA) and is subject to the same discretion based on RNA assay quality guidance.

#### Computational/predictive data-driven codes: PS1, PP3, BP1, BP4, BP7, and PM5

##### PS1

The original application of PS1 was intended for variants that cause the same protein effect as a previously established PV. Because *PALB2* has not yet been linked to missense pathogenicity, the application of PS1 to protein changes is not recommended. However, according to the SVI recommendations,^29^ the HBOP VCEP is employing the use of PS1 to different variants that cause the same RNA effect (ergo the same protein effect) with some precautions. The application and weighting of PS1 is in accordance with Table 2.

**Table 2:** PS1 code weights for variants with same predicted splicing event as known (likely) pathogenic variant

**Variant under assessment (VUA)**	**Baseline computational/predictive code applicable to VUA**	**Position of comparison variant relative to VUA**	**PS1 code applicable to VUA**	
			**with P comparison variant**	**with LP comparison variant**
Located outside splice donor/acceptor ±1,2 dinucleotide positions	PP3	same nucleotide	PS1	PS1_Moderate
	PP3	within same splice donor/acceptor motif (including at±1,2 positions)	PS1_Moderate	PS1_Supporting
Located at splice donor/acceptor ±1,2 dinucleotide positions	PVS1	within same splice donor/acceptor ±1,2 dinucleotide	PS1_Supporting	N/A
	PVS1	within same splice donor/acceptor region, but outside ±1,2 dinucleotide^a^	PS1_Supporting	PS1_Supporting
	PVS1_Strong, PVS1_Moderate, or PVS1_Supporting	within same splice donor/acceptor ±1,2 dinucleotide	PS1	N/A
	PVS1_Strong, PVS1_Moderate, or PVS1_Supporting	within same splice donor/acceptor region, but outside ±1,2 dinucleotide^a^	PS1_Moderate	PS1_Supporting

Prerequisite for all: the predicted event of the VUA must precisely match the predicted event of the comparison (likely) pathogenic variant (e.g., both predicted to lead to exon skipping, or both to lead to enhanced use of a cryptic splice motif, AND the strength of the prediction for the VUA must be of similar or higher strength than the strength of the prediction for the comparison [likely] pathogenic variant). For an exonic variant, predicted or proven functional effect of missense substitution(s) encoded by the VUA and (likely) pathogenic variant should also be considered before application of this code. Dinucleotide positions refer to donor and acceptor dinucleotides in reference transcript(s) used for curation. Designated donor and acceptor motif ranges should be based on position weight matrices for intron category (see methods). For GT-AG introns, these are defined as follows: the donor motif, last 3 bases of the exon and 6 nucleotides of intronic sequence adjacent to the exon; acceptor motif, first base of the exon and 20 nucleotides upstream from the exon boundary. Consider other motif ranges for non-GT-AG introns.

Reproduced, with permission, from Walker et al.^29^

a If relevant, splicing assay data for a pathogenic variant outside a ±1,2 dinucleotide position may be used to update a PVS1 decision tree and hence the applicable PVS1 code for a ±1,2 dinucleotide variant.

##### PP3/BP4

These codes are applied to *in silico* predictions of RNA or protein effects. Because missense pathogenicity is not currently established for *PALB2*, these codes are not applicable to protein effects. However, these codes may be applied to predicted RNA effects based on at least one well-established *in silico* predictor (e.g., SpliceAI^30^). Of note, splice predictors should be run on all variant types, where possible, including nonsense and frameshifts, which, on rare occasions, can still be prone to a splice defect that may rescue premature termination. The suggested cutoffs for PP3 and BP4 are ≥0.2 and ≤0.1, respectively.^29^ If observed RNA data are available and there *is* a splice defect (apply PVS1(RNA)), then PP3 or BP4 cannot also be applied. However, if there are no variant-specific impacts to splicing, you *can* apply PP3 or BP4 in conjunction with BP7(RNA) (Table 3). BP4 may also be applied in conjunction with BP7 (see BP7 below).

**Table 3:** Restrictions on combining criteria

	**PP3**	**PS1**	**PVS1**	**PVS1(RNA)**	**BP4**	**BP7**	**BP7(RNA)**
PS1	✓	–	–	–	–	–	–
PVS1	×	N/A	–	–	–	–	–
PVS1(RNA)	×	N/A	×	–	–	–	–
BP4	N/A	N/A	×	×	–	–	–
BP7	×	N/A	×	×	✓	–	–
BP7(RNA)	✓	N/A	×	×	✓	×	–

N/A, not applicable because the codes are unrelated; ✓, permitted; ×, not permitted; –, redundant with another cell.

##### BP1

BP1 is a code that is applied to missense variants when the primary mechanism of action of pathogenicity is through LoF. Based on published and unpublished functional studies, *PALB2* has a low rate of missense variants that are non-functional in relevant assays. True missense PVs are not yet confirmed or refuted but are thought to be rare. Given the very low likelihood that individual missense variants are pathogenic, this rule applies to all missense variants in *PALB2*.

##### BP7

BP7 can be applied to synonymous and deep intronic variants under the following conditions: deep intronic variants beyond (but not including) +7 on the donor side and −21 on the acceptor side. These boundaries have recently been justified by the SVI and are being adopted by this VCEP going forward.^29^ The former is based on the conservative sequence conservation of a donor site, while the latter is based on an attempt to capture the important branch site, which is critical to efficient splicing. If a well-established *in silico* predictor does not suggest a splice defect, BP4 can also be applied for cumulative evidence for LB. Although BP7 can be applied to any synonymous or deep intronic variant outside the boundaries set herein, it is not considered a conflicting piece of evidence if the other evidence purports a splice defect.

##### BP7_Variable(RNA)

The HBOP VCEP attempts to distinguish a predicted from an observed splice defect. As such, it has co-opted the use of BP7 by applying the extension (RNA) to describe a lack of observed splice effect. In cases where RNA data show no splice effect, use BP7_Variable(RNA) at a weight commensurate with the quality of the assay as described by the SVI.^29^

##### PM5

PM5 is a “hotspot” rule that supports the use of a pathogenic line of evidence to a variant at a codon where other known LP/P variants have occurred. Because of the lack of known missense pathogenicity for *PALB2*, this original concept is not applied. However, the HBOP VCEP applies PM5 as a supporting line of evidence (PM5_Supporting) to PVS1 or PVS1(RNA)-eligible variants that have a stop codon upstream of His1184. Of note, PM5_Supporting cannot be applied to predicted spliceogenic variants, nor can it be applied to observed RNA effects that have a weight less than very strong. This means that all variants with premature truncations upstream of His1184 are classified as at least LP with PVS1/PVS1(RNA) and PM5_Supporting. In the case where the variant is also rare—achieving PM2_Supporting—the variant will be classified as pathogenic (see Box 2). The application of this rule in this way is supported by the aforementioned lack of candidate rescue transcripts generated by alternative splicing for *PALB2* as well as the position of the C-terminal boundary at Tyr1183 (see PVS1). Of note, this rule applies to the position of the new stop codon and not the residues affected by the frameshift, such that variants that shift the reading frame through Tyr1183 but terminate N-terminally to Tyr1183 are not eligible for PM5_Supporting. For example, the pilot variant c.3543delT (p.Phe1181LeufsTer10) shifts the reading frame through Tyr1183, but the stop codon occurs at codon 1191 (a C-terminal extension) and is, therefore, not eligible for PM5_Supporting.

### Clinical data-driven specifications: PS4, PM3/BS2, and PP1/BS4

#### PS4

PS4 is a phenotype-driven rule that is intended for case-control studies supporting pathogenicity. In addition, a weaker (PS4_Moderate) “proband counting” method can substitute for a case-control study in instances where variants are too rare for statistically driven analyses. The monoallelic proband counting method requires that a phenotype be unique and rare enough such that its observation is a strong indicator of a specific disease. As previously discussed, breast, ovarian, and pancreatic cancers are relatively common, have multiple etiologies, and do not have sufficient features that distinguish genetic from sporadic disease. Thus, a proband counting method should not be employed unless it is carefully and statistically tailored to a specific cohort.^12^^,^^13^ Case-control analyses, however, are eligible for PS4 when statistically significant (*p* ≤ 0.05) and when the odds ratio, relative risk, or hazard ratio is ≥3 and the lower 95% confidence interval (CI) is ≥1.5.

#### PM3 and BS2

As bi-allelic PVs in *PALB2* cause FA-N, an exceedingly rare, early-onset, autosomal recessive disease, the observation of multiple variants in affected or confirmed unaffected individuals is informative under the PM3 or BS2 codes, respectively (Table 4). Given the strength of these codes and the ascertainment bias in the literature reporting on the most severe instances of individuals with FA, the HBOP VCEP has aligned with the BRCA1/2 VCEP and applied limitations to the use of these codes in an effort to be conservative and protect against possible instances of individuals with subclinical FA.^13^ Toward PM3, the variant under assessment (VUA) must be sufficiently rare (<0.01%) so as not to risk co-occurring with another variant by chance based on frequency. In addition, given the large number of genes associated with FA, it is also advisable to consider whether the individual has been molecularly subtyped and/or had other causes for FA ruled out by panel or exome testing. Multiple affected (unrelated) individuals are additive within the point scheme in Table 4. Additionally, the VCEPs have outlined guidance for what constitutes an individual whose phenotype is consistent with FA (Box 1):

**Table 4:** PM3 and BS2 bi-allelic code strengths

**PM3**^a^		**Points per unrelated FA-N proband (PM3)**^f^		
	**classification/zygosity of the co-occurring variant**	**(likely) pathogenic in *trans* or homozygous**^c^	**phase unknown**	
	**phenotype consistent**^b^	**2**	**1**	
BS2		Points per proband meeting age/cancer diagnosis criteria^g^		
	proband presentation	VUA in *trans* with (likely) pathogenic in same gene^d^	Homozygote^e^	Phase unknown, co-occurs with (likely) pathogenic in same gene
	first cancer onset >50 years or cancer-unaffected at age at last follow-up of >50 years	4	2	1
	first cancer onset 40–50 years or cancer-unaffected at age at last follow-up of 40–50 years	2	1	0.5

a Variant under assessment must be sufficiently rare (not meeting a benign population evidence code). Consider other gene panel test results as potential explanation for phenotype.

b Phenotype consistent with *PALB2*-related Fanconi anemia (FA) (see Box 1).

c Co-occurrent (likely) pathogenic variant should be assigned classification using VCEP specifications. Stipulation for in *trans* can be met by genotyping of at least one parent, or assumed if VUA is seen with at least two different (likely) pathogenic variants. Further, co-occurrence in *trans* can be inferred in a homozygous FA-affected individual due to a consanguineous union, or if both maternal and paternal lineages present with family history of cancer consistent with *PALB2* related cancers.

d VUA, variant under assessment. VUA should not be bioinformatically predicted (or experimentally proven) to have a clinically important effect on protein or mRNA splicing. Co-occurrent (likely) pathogenic variant should be assigned classification using VCEP specifications. Stipulation for in *trans* can be met by genotyping of at least one parent, or assumed if VUA is seen with at least two different P/LP variants.

e Apply only for phenotyped individuals from clinical or research cohorts. Not to be applied for data used to assign frequency-based codes.

f Point totals for PM3: 1 = supporting, 2 = moderate, 4 = strong, ≥8 = very strong.

g Point totals for BS2: 1 = supporting, 2 = moderate, ≥4 = strong.

Box 1 **Fanconi anemia phenotype**

Fanconi anemia (FA) of any subtype is generally considered an exceedingly rare, severe, early-onset disease with variable features. In the case of *BRCA2*, individuals with hypomorphic FA variants have been described who are diagnosed at older ages with less severe phenotypes. The criteria set forth in Table 4 are designed to accommodate such hypomorphs and are recommended to be applied to all FA-associated genes that may not be as well described due to the extreme infrequency of their identification and due to ascertainment bias (for severe phenotype) in the literature.

(1) General considerations
  • Variant may not exceed general population frequency >0.01%
  • Consider other gene panel test results as potential explanation for phenotype
  • Multiple unrelated individuals are additive
(2) Phenotype consistent
  • Chromosomal breakage with 1 clinical feature -OR-
    ⁃ Example: chromosomal breakage testing + microcephaly/triangular face
  • At least two of three clinical features from separate categories without chromosomal breakage studies
    ⁃ Example (without chromosomal breakage): myelodysplastic syndrome and microcephaly/triangular face
(3) Positive for chromosome breakage test
  • Increased chromosome breakage and/or radial forms on cytogenetic testing of lymphocytes with diepoxybutane (DEB) or mitomycin C (MMC)
(4) Clinical features indicative of FA, including
  • Physical features (in ∼75% of affected persons), including
  • Prenatal and/or postnatal short stature
  • Abnormal skin pigmentation (e.g., café-au-lait macules, hypopigmentation)
  • Skeletal malformations (e.g., hypoplastic thumb, hypoplastic radius)
  • Microcephaly, triangular face
  • Ophthalmic anomalies
  • Genitourinary tract anomalies
  • See Orphanet for full list of >100 HPO terms (and their reported frequency)
(5) Pathology findings and laboratory findings (non-cancer related) include
  • Progressive bone marrow failure (unrelated to cancer treatment)
  • Aplastic anemia
  • Myelodysplastic syndrome
  • Exceptionally severe toxicity from chemotherapy (e.g., severe neutropenia) or radiation
  • Macrocytosis
  • Cytopenia (especially thrombocytopenia, leukopenia, and neutropenia)
  • Increased fetal hemoglobin (often precedes anemia)
(6) Cancer diagnosis ≤5 years, particularly
  • Blood cancers (acute myeloid leukemia)
  • Brain cancers (medulloblastoma, neuroblastoma)
  • Wilms tumor

Specifications are adapted from definitions from GeneReviews (last revision June 3, 2021)

In the benign direction, the weight afforded decreases based on a confirmed *trans* occurrence, a homozygous occurrence, or a phase-unknown occurrence. In addition, the weight afforded decreases if an individual is diagnosed with cancer between age 40 and 50 years old. Both of these caveats protect against downgrading variants that may be pathogenic but causing subclinical or late-onset disease (see Table 4). Because the phenotype and age of these individuals are not consistently or verifiably disclosed in general population databases, homozygous individuals from these sources are not used for BS2.

#### PP1 and BS4

PP1 and BS4 are codes that describe the co-segregation (or lack thereof) of a variant with disease. Because *PALB2* demonstrates incomplete penetrance, the HBOP VCEP requires the use of a statistical model for ascertaining co-segregation, including Bayes factor or logarithm of the odds (LOD) scores.^31^^,^^32^ The HBOP VCEP does not permit the use of “meiosis counting” as can be adopted for more pathognomonic and rare diseases.^33^ Penetrance should be set according to Yang et al.,^6^ and weights should be applied as follows.

(1) PP1_Strong: LOD ≥ 1.26 or Bayes factor (likelihood ratio [LR]) ≥18:1
(2) PP1_Moderate: LOD ≥ 0.60 or Bayes factor (LR) ≥4:1
(3) PP1: LOD ≥ 0.3 or Bayes factor (LR) ≥2:1
(4) BS4_Supporting: LOD ≤ −0.32 or Bayes factor (LR) ≤0.48
(5) BS4_Moderate: LOD ≤ −0.64 or Bayes factor (LR) ≤0.23
(6) BS4: LOD ≤ −1.28 or Bayes factor (LR) ≤0.053:1

For co-segregation of a bi-allelic genotype in multiple related individuals with FA, the HBOP VCEP proposes to apply PP1 as set forth by other VCEPs such as the Glanzmann thrombasthenia VCEP (https://cspec.genome.network/cspec/ui/svi/doc/GN011). In a family where the genotype does not segregate with disease, consider applying BS2.

### Modified evidence code combinations

The HBOP VCEP largely adopted the categorical evidence code combinations published by Richards et al. in 2015^1^ with two modifications. To achieve a minimum LP classification for PVS1-eligible variants, the combination of PVS1 plus one additional supporting line of pathogenic evidence is allowed to achieve LP (see discussion point in PM5). In addition, one strong line of evidence in the benign direction is sufficient to achieve an LB classification. Both of these specific modifications are in line with a Bayesian model of variant interpretation published by the SVI.^10^ Box 2 outlines specific code-weight combinations for final classifications.

Box 2 **Code combinations for classification**

Pathogenic criteria

(1) Pathogenic
  (a) 1 very strong -AND-
    (i) ≥1 strong -OR-
    (ii) ≥2 moderate -OR-
    (iii) 1 moderate -AND- 1 supporting -OR-
    (iv) ≥2 supporting
  (b) ≥2 strong -OR-
  (c) 1 strong -AND-
    (i) ≥3 moderate -OR-
    (ii) 2 moderate -AND- ≥2 supporting -OR-
    (iii) 1 moderate -AND- ≥4 supporting
  (d) 1 moderate -AND- ≥4 supporting
(2) Likely pathogenic
  (a) 1 very strong -AND-
    (i) 1 moderate -OR-
    (ii) 1 supporting
  (b) 1 strong -AND-
    (i) 1–2 moderate -OR-
    (ii) ≥2 supporting
  (c) ≥3 moderate -OR-
  (d) 2 moderate -AND- ≥2 supporting -OR-
  (e) 1 moderate -AND- ≥4 supporting

Benign criteria

(1) Benign
  (a) 1 standalone (BA1) -OR-
  (b) ≥2 strong (BS1–BS4)
(2) Likely benign
  (a) 1 strong -OR-
  (b) 1 strong -AND- 1 supporting -OR-
  (c) ≥2 supporting

### Pilot

**Figure 2:**
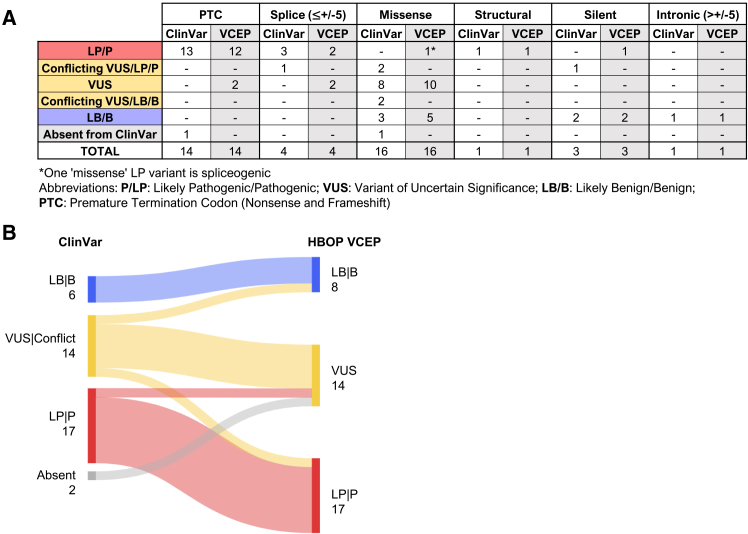
*PALB2* pilot variant categorization (A) Thirty-nine pilot variants are displayed as community classification in ClinVar (left), where VUS/LP/P conflicting interpretation variants and VUS/LB/B conflicting interpretation variants are binned along with consensus VUS as “ClinVar VUS/Conflict.” Interpretation with the HBOP rules specifications for *PALB2* are on the right. Granular details of the type of conflict and the type of variant are presented in Table S2. (B) A Sankey plot demonstrates the transition of individual variants as classified in ClinVar (left) versus classified by the HBOP VCEP (right) into likely benign/benign (LB/B in blue), VUS or conflicting (yellow), and likely pathogenic/pathogenic (LP/P in red) categories. Two variants were not in ClinVar and are in gray.

To arrive at the final iteration of the specifications for *PALB2*, the HBOP VCEP used them to classify a group of 39 pilot variants: nine frameshift, five nonsense, one gross deletion, five intronic, 16 missense, and three synonymous variants (Table S2). This group included a variety of variant types that were broadly representative of classification types deposited in ClinVar. Six variants had a consensus ClinVar classification of LB/B, and the HBOP VCEP classified all six as LB/B. Among the 17 ClinVar consensus LP/Ps, the HBOP VCEP also classified 15 as LP/P (Figure 2).

The remaining two variants, *PALB2* c.3543del (p.Phe1181LeufsTer10) and *PALB2* c.1684+1G>A, were classified as VUS by the HBOP VCEP. The frameshift only received PVS1_Strong, instead of very strong, due to the PTC occurring after the C-terminal boundary of Tyr1183. The intronic variant also received PVS1_Strong instead of very strong due to the in-frame impact on a region with no known clinically relevant function (list D in Table S1). Of the 14 variants that were VUS or conflicting (VUS plus other classifications), the HBOP VCEP also classified ten as VUS. Two ClinVar VUS/conflicts (*PALB2* c.3350G>A [p.Arg117Lys] and *PALB2* c.2559C>T [p.Gly853=]) were classified by the HBOP VCEP as LP/P, likely due to how the observed RNA data were weighted and/or the modified code combinations allowing PVS1 + 1 supporting pathogenic line of evidence to equate LP (see Box 2). Lastly, two ClinVar VUS/conflicts (*PALB2* c.3054G>C [p.Glu1018Asp] and *PALB2* c.3249G>C [p.Glu1083Asp]) were classified by the HBOP VCEP as B. While it is not always granularly transparent why a variant is a VUS in ClinVar, it is likely that the discrepancy is due to the VCEP’s established BA1 threshold and the broad applicability of BP1 for missense variants, which other depositors may not have adopted. Two pilot variants were absent from ClinVar and were both classified as VUS by the HBOP VCEP (Table S2). The pilot variants were deposited into ClinVar.^34^

Among the 23 non-VUS-classified variants in ClinVar, only two variants differed, and both were the result of a more conservative application of the code PVS1 resulting in a VUS from the VCEP compared to an LP/P consensus in ClinVar. Among the 14 ClinVar VUS/conflicting variants, the HBOP VCEP was able to resolve four of them: two as B (both with BA1, BS2, and BP1 applied at variable strength) and two as LP/P (both with PVS1(RNA) applied). Unsurprisingly, the remaining VUS are C-terminal truncations (*n* = 2), missense variants (*n* = 10), or splicing variants with in-frame effects (*n* = 2).

## Discussion

The HBOP VCEP has developed a conservative set of variant interpretation guidelines that are specific to *PALB2*. Two main features limit the application of many codes: (1) the commonality of HBOP cancers and (2) the dearth of evidence supporting missense as a mechanism of pathogenicity. Though they may exist, their putative extreme rarity makes it difficult to identify them and validate them as LP/P. Despite this conservative application of evidence codes, the pilot study demonstrated that the variant interpretation community largely agrees with these challenges, as none of the pilot missense variants in ClinVar were classified as LP/P. The pilot also demonstrated that there was 84% (31/37) concordance in the interpretations in ClinVar with the HBOP VCEP interpretation. Among the variants that were VUS or conflicting in ClinVar, the VCEP was able to resolve 29% (4/14); however, the VCEP took a more conservative stance and classified two ClinVar LP/P as VUS. In total, the VCEP achieved 25/37 non-VUS classifications while ClinVar reflected 23/37 non-VUS/conflicting classifications, demonstrating a 5% increase in certainty of classifications from the VCEP. Thus, these guidelines are simultaneously more conservative but more effective for the classification of *PALB2* variants.

Given this positive outcome, the HBOP VCEP has established a group of biocurators comprising a professional curator as a lead and several ClinGen volunteer biocurators who are trained for *PALB2*-specific variant interpretation to continue the work of *PALB2* variant classification and deposit to ClinVar. As per ClinGen protocol, the prioritization of variants is based on conflicting classifications in ClinVar as well as variants of special interest that may be submitted by the community. Currently, ClinVar contains a total of ∼6,300 *PALB2* variants (https://www.ncbi.nlm.nih.gov/clinvar, accessed May 31, 2025), approximately 44% of which are VUSs (2,762 variants) and another ∼9% (597 variants) of conflicting interpretations. Among the VUSs, 90% are missense variants (2,491 missense VUSs/2,762 total VUSs). This highlights the critical need to gain clarity on the role of missense variation in pathogenicity that may lead to more refined guidelines that are better equipped to resolve these missense VUSs.

Toward that end, we are aware that *PALB2* continues to be heavily studied, and new data will be forthcoming that will influence the specifications set forth herein. Specifically, the HBOP VCEP anticipates that generation and calibration of high-throughput functional data for use in variant curation will influence the specification and use of functional and protein *in silico* codes. The group will meet to discuss any new data and how they should influence the specifications. Updated guidelines will be submitted to the SVI for approval and updated accordingly on ClinGen websites (https://cspec.genome.network/cspec/ui/svi/doc/GN077). Adoption of the specifications described herein, as well as continuing VCEP-driven variant interpretation and review, will help to improve harmonization across interpretations, enhancing clinical practice in the hereditary cancer space.

## Data and code availability

The published article includes all datasets generated or analyzed during this study. The accession numbers for the variant interpretations reported in Table S2 are ClinVar. Any genotype data that is not in the public domain can be requested from the corresponding authors and/or the VCEP Chairs or Coordinator identified here: https://clinicalgenome.org/affiliation/50039/. The VCEP will forward requests on to the original submitter.

## Acknowledgments

The HBOP VCEP is supported, in part, by U24CA258058 (A.B.S., M.F.H.B., M.A.H., F.J.C., and M.d.l.H.). Other members report the following funding: Spanish Ministry of Science and Innovation and Acción Estratégica en Salud 2024, ISCIII (PI24/00267)/FEDER from Regional Development European Funds (M.d.l.H.); NHMRC Investigator Fellowship (APP177524) (A.B.S.); 10.13039/501100000289Cancer Research UK (EDDPGM-Nov22/100004) (C.T.); 10.13039/501100018956NIHR Cambridge Biomedical Research Centre (NIHR203312) (M.T.); 10.13039/100000002NIH-R01-CA264971 (S.V.T.); NIH-ES031796, DoD CDMRP (BC201356), and ACS Discovery Boost (DBG-24-1243614-01-DMC) (K.A.B.); 10.13039/501100000024CIHR project grant PJT-517664 (J.-Y.M.); 10.13039/100001006Breast Cancer Research Foundation (A.N.M.); New Zealand Health Research Council (#22/187) (L.C.W.); 10.13039/501100000024Canadian Institutes of Health Research (FDN-148390) (W.D.F.); and 10.13039/100000002NIH (R35CA253187 and P50CA116201) and the 10.13039/100001006Breast Cancer Research Foundation (F.J.C.). We thank Melissa Cline for contributions to acquiring VCEP funding and planning software development. We thank all past HBOP VCEP members for additional contributions.

## Declaration of interests

M.E.R., T.B., T.P., and C.C.Y. were paid employees of Ambry Genetics. L.Z. was a paid employee of Natera. S.H. was a paid employee of GeneDx.

## Web resources

ClinVar, https://www.ncbi.nlm.nih.gov/clinvar
CSpec registry, guidelines for PALB2, https://cspec.genome.network/cspec/ui/svi/doc/GN077
CSpec registry, MONDO ID for Glanzmann thrombasthenia, https://cspec.genome.network/cspec/ui/svi/doc/GN011
ENIGMA BRCA1 and BRCA2 VCEP, https://clinicalgenome.org/affiliation/50087/
HBOP VCEP, https://clinicalgenome.org/affiliation/50039/
OMIM, https://www.omim.org/
PALB2 in gnomAD, https://gnomad.broadinstitute.org/gene/ENSG00000083093?dataset=gnomad_r4
SVI recommendation for absence rarity, https://clinicalgenome.org/site/assets/files/5182/pm2_-_svi_recommendation_-_approved_sept2020.pdf

## Supplemental information

Table S1. PVS1 decision tree

Table S2. Pilot details
